# Higher Serum-Soluble *α*-Klotho Level Does Not Predict Longer Survival after Stroke

**DOI:** 10.1155/2020/9283651

**Published:** 2020-12-07

**Authors:** Dagmara Adamska-Tomaszewska, Jarosław Wajda, Katarzyna Wyskida, Dawid Bednarczyk, Maciej Świat, Aleksander J. Owczarek, Monika Puzianowska-Kuźnicka, Magdalena Olszanecka-Glinianowicz, Jerzy Chudek

**Affiliations:** ^1^Department of Pathophysiology, Medical Faculty in Katowice, Medical University of Silesia, Katowice, Poland; ^2^Dialysis Center in Rybnik, Regional Specialist Hospital No. 3 in Rybnik, Rybnik, Poland; ^3^Health Promotion and Obesity Management Unit, Department of Pathophysiology Medical Faculty in Katowice, Medical University of Silesia, Katowice, Poland; ^4^Department of Neurology with Stroke Unit, Regional Specialist Hospital No. 2 in Jastrzębie-Zdrój, Jastrzębie-Zdrój, Poland; ^5^Department of Neurology with Stroke Unit, Regional Specialist Hospital No. 3 in Rybnik, Rybnik, Poland; ^6^Jan Długosz University in Częstochowa, Częstochowa, Poland; ^7^Department of Statistics, Department of Instrumental Analysis, Faculty of Pharmaceutical Sciences in Sosnowiec, Medical University of Silesia, Katowice, Poland; ^8^Department of Human Epigenetics, Mossakowski Medical Research Centre, Polish Academy of Sciences, Warsaw, Poland; ^9^Department of Geriatrics and Gerontology, Medical Centre of Postgraduate Education, Warsaw, Poland; ^10^Pathophysiology Unit, Department of Pathophysiology Medical Faculty in Katowice, Medical University of Silesia, Katowice, Poland; ^11^Department of Internal Medicine and Oncological Chemotherapy, Medical Faculty in Katowice, Medical University of Silesia, Katowice, Poland

## Abstract

**Results:**

There were 5 recurrent strokes and 89 deaths during the 36-month follow-up. Even though no significant differences in OS and SFS between soluble *α*-Klotho level tertile groups were recorded, unexpectedly, OS and SFS were highest in patients with the lowest soluble *α*-Klotho concentrations. Moreover, the Cox proportional models adjusted for established risk factors, kidney function, and the severity of stroke revealed that each 100 pg/mL increase in soluble *α*-Klotho levels was associated with decreased OS (HR = 0.951 (0.908–0.995), *p* < 0.05) and SFS (HR = 0.949 (0.908–0.993), *p* < 0.05). In addition, the *α*-Klotho to iFGF23 index was predicting neither OS nor SFS.

**Conclusion:**

Soluble *α*-Klotho levels in serum were not related to the severity of neurological deficits and long-term outcomes in patients with IS. No neuroprotective effect of soluble *α*-Klotho levels in patients with IS was demonstrated.

## 1. Background

The aim of this study was to analyze the association between serum concentration of soluble *α*-Klotho and the clinical outcome in patients with ischemic stroke (IS) including overall survival (OS) and stroke-free survival (SFS) during a 36-month follow-up.

## 2. Introduction

Ischemic stroke (IS) is a major cause of disability and one of the major causes of mortality in the aging human population [1]. Hypertension, smoking, obesity, type 2 diabetes, dyslipidaemia, and low physical activity levels explain, to a large extent, early development of atherosclerosis and increasing incidence of IS. Also, other factors predisposing to vascular aging may affect the occurrence of IS and, potentially, its outcome.

Two decades ago, Kuro-o et al. [2] described a Klotho-deficient mice model of aging, with extensive medial calcification of the aorta and intimal thickening in middle-sized muscular arteries as a part of the phenotype associated with shortened lifespan.

Klotho, a transmembrane protein, is predominantly but not exclusively expressed in the distal tubular cells of the kidney, parathyroid glands, and choroid plexus of the brain. Its soluble form is created mostly by proteolytic shedding of the extracellular part of the full-length Klotho [3]. In the kidney, *α*-Klotho plays a role of a coreceptor for fibroblast growth factor 23 (FGF23) associated with arteriosclerosis prevention [4] and is involved in phosphorus excretion [5], whereas soluble *α*-Klotho participates in the regulation of endothelial nitric oxidase synthase (eNOS) activity and calcium channel transient receptor potential vanilloid 5 (TRPV5) in calcium homeostasis [6].

Serum-soluble *α*-Klotho levels correlate negatively with the prevalence of arteriosclerosis [7], which suggests its preventive role in the development of premature arteriosclerosis [8], potentially related to the enhanced synthesis of NO by the endothelium [9] and suppression of TNF-*α*-induced expression of adhesion molecules [5].

Indeed, Woo et al. [10] described a negative association between soluble plasma *α*-Klotho levels and the presence, burden, and progression of cerebral small vessel disease (high-grade white matter hyperintensities in periventricular white matter) in brain magnetic resonance imaging in patients with IS. In addition, subjects after IS with higher serum-soluble *α*-Klotho levels tend to recover better after the incident [11]. This is in line with the improved neurobehavioral deficits and inhibition of postischemia neuronal loss in the hippocampal and caudate putamen subregions in mice with the overexpression of Klotho [12].

As recovery after IS remains the point of the highest interest, we assessed soluble *α*-Klotho and intact FGF23 (iFGF23) and tested whether an association exists between *α*-Klotho serum levels and the soluble *α*-Klotho/iFGF23 index and the long-term IS outcomes, such as overall survival and stroke-free survival. Till now, the neuroprotective properties of soluble *α*-Klotho levels in humans were shown only in a short-term observation [11].

## 3. Patients and Methods

The prospective study included 217 of 240 consecutive patients with the onset of symptoms of IS within the last 24 hours before the admission, diagnosed according to the WHO criteria [13] and based on radiological images (computed tomography (CT) and/or magnetic resonance (MRI) of the head). All patients were hospitalized during the acute phase of IS in the Department of Neurology of the Provincial Hospital in Rybnik and diagnosed and treated in accordance with the current guidelines [14]. The most probable mechanism of stroke was established according to the TOAST classification (Trial of ORG 10172 in Acute Stroke Treatment) [15]. Patients with history of cancer, apparent inflammation, and impairment in activities of daily living (ADL) before the IS diagnosis were initially excluded. In addition, from the cohort of 240 included patients, we have excluded 23 patients with other classified (*N* = 8) and unclassified (*N* = 15) strokes. The clinical course of IS was assessed on the basis of two main criteria: the severity of neurological deficit evaluated according to the NIHSS (National Institute of Health Stroke Scale) and functional status at admission and discharge evaluated with the modified Rankin Scale (mRS).

The deep-frozen serum originated from blood obtained at admission to the Stroke Unit between January 2013 and August 2015. The approval for the utilization of serum samples and retrieval of data from medical reports without an informed consent from IS patients was granted by the Bioethics Committee of the Medical University of Silesia in Katowice, which allowed inclusion of unconscious patients, thus preventing a selection bias.

The data retrieved from medical records included the following: clinical status, comorbidities, medication, previous ischemic cerebrovascular episodes, cardiovascular risk factors, and routinely evaluated laboratory parameters (total blood count, serum glucose, creatinine, total cholesterol, LDL cholesterol, HDL cholesterol, triglycerides levels, and urine analysis).

Complete follow-up data for mortality were obtained from the Registry Office (as for May 30th, 2017).

### 3.1. Measurements

Nonroutine assessments were performed in the Laboratory of the Department of Pathophysiology, Medical University of Silesia in Katowice.

Commercially available ELISA kits were used for assessment of serum concentrations of soluble *α*-Klotho levels (Immuno-Biological Laboratories Co. Ltd., Fujioka-Shi, Gunma, Japan) and iFGF23 (Immutopics, San Clemente, CA, USA), with the mean intra- and interassay coefficients <3% and <6.5% (soluble *α*-Klotho) and <4.4% and <6.1% (iFGF23). Serum intact parathyroid hormone (iPTH), phosphorus, and calcium levels were assessed using commercially available kits on the Cobas E411 and Cobas 111 analyzers (Roche Diagnostics GmbH, Mannheim, Germany) with interassay coefficients of variability <6.5%, <2.3%, and <1.3%, respectively.

### 3.2. Data Analysis

Glomerular filtration rate (eGFR) was estimated according to the short MDRD (Modification of Diet in Renal Disease) formula [16]. Patients with eGFR < 60 mL/min/1.73 m^2^ or albuminuria in a routine urine analysis were considered as having chronic kidney disease.

We have calculated soluble *α*-Klotho to the iFGF23 index in order to assess the mutual proportion between serum concentration of coreceptor (*α*-Klotho) and agonist (FGF23) of FGF23 receptor.

The end points of the analysis were 12, 24, and 36 months of overall survival (OS) and stroke-free survival (SFS) after the IS.

### 3.3. Statistical Analysis

Statistical analysis was performed using STATISTICA 13.0 PL (TIBCO Software Inc., Palo Alto, CA, U.S.), StataSE 13.0 (StataCorp LP, TX, U.S.), and R software. Statistical significance was set at a *p* value below 0.05. All tests were two-tailed. Imputations were not done for missing data. Nominal and ordinal data were expressed as percentages. Interval data was expressed as mean value ± standard deviation in the case of normal distribution. In the case of data with skewed or nonnormal distribution, it was expressed as median, with lower and upper quartiles. Distribution of variables was evaluated by the Anderson-Darling test and the quantile-quantile (Q-Q) plot. Homogeneity of variances was assessed by the Levene test.

In order to show the survival rate and cumulative hazard estimates according to the follow-up time, the Kaplan-Meier curves estimates were constructed. These were used with a log-rank test to compare survival distribution between two subgroups. Risk factors of death as well as composite end points (death or a recurrent stroke) were analyzed with univariable and multivariable stepwise backward Cox proportional hazard regression. Schoenfeld residuals were used to test proportional hazard (PH) assumption. The concordance probability, which is defined as the probability that predictions and outcomes are concordant, was calculated with Gönen and Heller's *K* concordance coefficient. The extended mean was obtained by extending the Kaplan-Meier product-limit survivor curve. This was extended to zero by using an exponentially fitted curve and then computing the area under the entire curve.

## 4. Results

**Figure 1:**
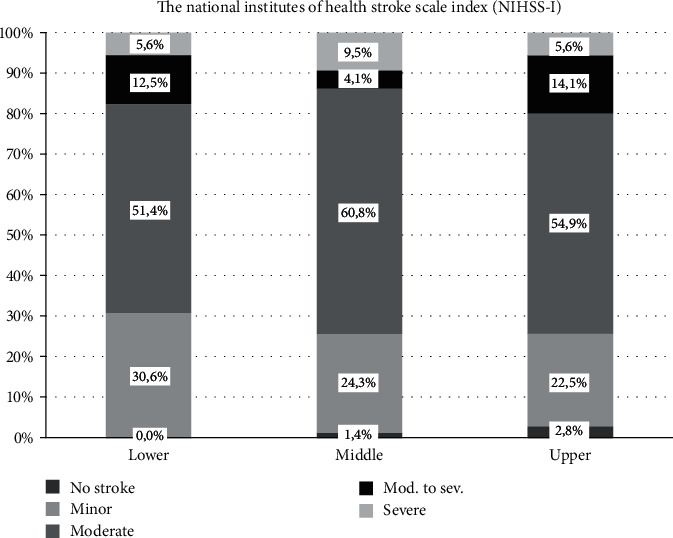
The severity of stroke according to NIHSS-I score in serum-soluble *α*-Klotho level tertiles.

Detailed characteristics of 217 patients (99 men and 118 women) grouped into three serum-soluble *α*-Klotho concentration tertiles are shown in Table 1. The mean ages of these subgroups were 72 ± 11, 72 ± 12, and 71 ± 11 years. The subgroups were comparable with respect to concomitant disease burden (except of the obesity), medication usage prior to IS, and basic laboratory findings. The IS types were similar among the groups. In addition, there were no differences in the severity of IS according to NIHSS-I score results between soluble *α*-Klotho level tertiles (Figure 1).

No significant differences were found in serum iFGF23 between the subgroups, while the values of the soluble *α*-Klotho/iFGF23 index were incising proportionally to the levels of soluble *α*-Klotho in the subsequent tertiles. There was no correlation between the CRP and serum iFGF23, soluble *α*-Klotho, and *α*-Klotho/iFGF23 index.

**Figure 2:**
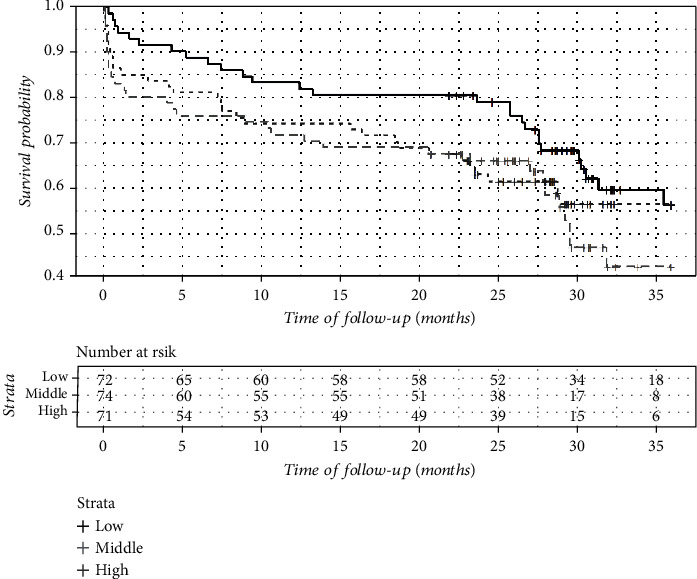
Overall survival (OS) in serum-soluble *α*-Klotho level tertiles (Kaplan-Meier plot).

**Figure 3:**
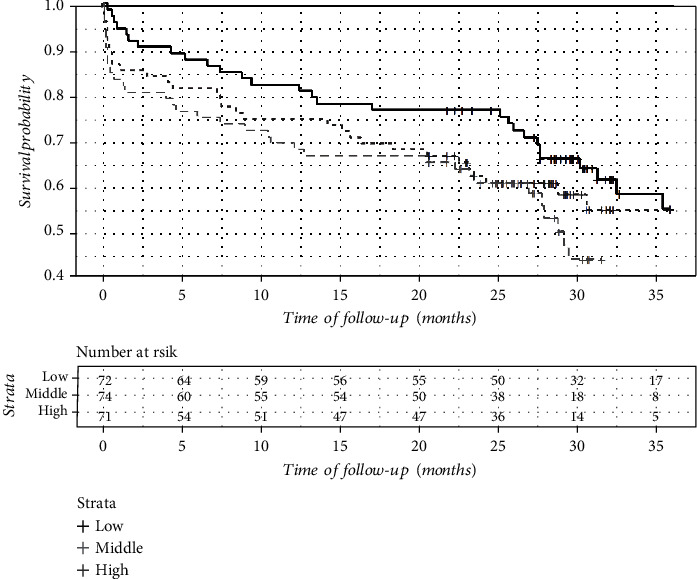
Stroke-free survival (SFS) in serum-soluble *α*-Klotho level tertiles (Kaplan-Meier plot).

There were 5 recurrent strokes and 89 deaths including 19 in-hospital fatal outcomes during the 36-month follow-up. The OS and SFS were highest in the lowest soluble *α*-Klotho concentration tertiles (Figures 2 and 3), but these differences were not significant (Table 1).

Survivors were significantly younger, had better kidney excretory function (eGFR), higher concentrations of LDL cholesterol and triglycerides, and lower prevalence of atrial fibrillation and experienced less severe IS according to NIHSS-I. However, no difference in serum iFGF23, soluble *α*-Klotho, and the *α*-Klotho/iFGF23 index was noted between survivors and those who died (Table 2).

The Cox proportional models revealed that each 100 pg/mL increase in soluble *α*-Klotho levels was associated with decreased OS (HR = 0.940 (95% CI: 0.894–0.988), *p* < 0.05) and SFS (HR = 0.935 (0.890–0.981), *p* < 0.01). After adjustment for age, obesity, type 2 diabetes, hypertension, atrial fibrillation, smoking, LDL cholesterol, eGFR, and severity of stroke according to the NIHSS-I score, the difference remained significant for OS (HR = 0.951 (0.908–0.995), *p* < 0.05) and SFS (HR = 0.949 (0.908–0.993), *p* < 0.05).

The soluble *α*-Klotho to iFGF23 index was not a predictor of OS (crude HR = 0.999 (95% CI: 0.993–1.006), *p* = 0.89, and adjusted for age, obesity, type 2 diabetes, hypertension, atrial fibrillation, smoking, LDL cholesterol, eGFR, and NIHSS-I score HR = 0.999 (95% CI: 0.993–1.005), *p* = 0.67) or SFS (crude HR = 1.001 (95% CI: 0.996–1.007), *p* = 0.69, and adjusted for age, obesity diabetes, hypertension, atrial fibrillation, smoking, LDL cholesterol, eGFR, and NIHSS-I score HR = 0.999 (95% CI: 0.994–1.005), *p* = 0.96).

## 5. Discussion

The correlation between arteriosclerosis and cardiovascular events such as myocardial infractions has been found a long time ago. More recently, a spectrum of established risk factors (e.g., abnormally high cholesterol levels, hypertension, type 2 diabetes, smoking, and obesity) has been extended to include new potential factors such as soluble *α*-Klotho levels, based on a 6-year observation by Memmos et al. [17] of a cohort of 804 older adults who showed that individuals with *α*-Klotho level in the lowest tertile (<575 pg/mL) had an increased risk of death (HR = 1.78, 95% CI 1.20–2.63) compared with participants in the higher quartile (>763 pg/mL) [18]. Similarly, Otani-Takei et al. [19] and Memmos et al. [17] showed higher cardiovascular risk in haemodialysis patients with low soluble *α*-Klotho levels. However, more recently, a multicenter European study failed to find a predictive significance of soluble *α*-Klotho levels in 2948 patients referred for coronary angiography during an almost 10-year follow-up [20].

We did not find a neuroprotective effect of soluble *α*-Klotho levels in a cohort of 217 patients with IS. Even though patients divided according to the soluble *α*-Klotho level tertiles had similar demographic and pharmacotherapy characteristics, IS types, and coexisting diseases (except obesity), both the severity of IS and long-term (12, 24, and 36 months) outcomes were similar. Our findings are not in line with experimental model described by Zhu et al. [3], showing the inhibition of postischemia neuronal loss by the overexpression of Klotho. Moreover, in the study performed by Lee et al. [21], patients with lower soluble *α*-Klotho levels had greater cerebral infarction volume and neurological deficit, and this fact seems to explain the worse 3-month outcome in their cohort. We did not measure the cerebral infarction volume; therefore, we cannot compare this parameter between the two cohorts. In addition, our cohort was older by almost 10 years. An association between *KL-VS* functional genetic variants of *KLOTHO* and early-onset IS was found in patients younger than 40 years [8]. However, in older adults, this genetic predisposition may be dominated by lifestyle-related factors predisposing to the development of atherosclerosis and heart arrhythmias, a strong risk factor for IS. This can be a reason why, in our patients, there was no association between soluble *α*-Klotho levels and the severity of neurological deficit assessed according to the NIHSS.

It is worth mentioning that other reports outside the neurology field showed unfavorable effect of increased soluble *α*-Klotho levels. For example, an increase in soluble *α*-Klotho in patients with septic shock was independently associated with higher mortality [22]. It cannot be excluded that the predictive value of soluble *α*-Klotho levels, demonstrated in septic patients with chronic kidney disease [23], may result from increased cleavage and shedding of membrane bound Klotho via upregulation of disintegrin and metalloproteinase domain-containing protein 10 (ADAM10) in severe sepsis and septic shock [24]. Another disease directly related to inflammation is liver cirrhosis. It was observed that levels of soluble *α*-Klotho in the plasma of heavy alcoholics with cirrhosis were significantly higher than those of healthy controls [25]. In our study, the severity of inflammation evaluated by the CRP levels was much lower, and the activity of ADAM10 was unaltered. In consequence, we did not find an association between CRP and soluble *α*-Klotho levels.

Moreover, a study of 443 nursing home residents showed that an isolated measure of the plasma-soluble *α*-Klotho level was not associated with mortality at 24 months [26]. Therefore, evidence for association of *α*-Klotho levels with longevity in humans is still lacking.

The above-cited studies preclude the conclusion that soluble *α*-Klotho has universal protective properties in all clinical states. It rather seems that a number of factors may affect its concentration and affect its predictive role in various clinical conditions, including IS.

The question how Klotho, a membrane coreceptor for the phosphaturic hormone FGF23 that affects intracellular calcium homeostasis and synthesis of nitric oxide by endothelial cells, may affect the IS outcome is mainly speculative. In Klotho-deficient mice, an aging-like phenotype was related to altered mineral-ion homeostasis linked to the increased synthesis of active vitamin D (hypervitaminosis) [27]. However, in the human population, the observed variability in soluble *α*-Klotho levels does not affect phosphate-calcium homeostasis, as shown also in our study. A study assessing the role of Klotho isoforms showed that 130 kDa soluble Klotho plays a stimulatory role in cardiac myofibroblast growth and activity through the FGF pathway, whereas 65 kDa isoform exerts an antifibrotic effect in cardiac myofibroblasts [28]. Therefore, interpretation of our results and findings of others which are based on total soluble *α*-Klotho levels should be cautious, as *α*-Klotho isoforms seem to have different biological functions.

Our study has some limitations. First, we did not analyze Klotho variants and the size of cerebral infarction volumes. Furthermore, the number of recurrent strokes was very low, and the size of our study group was underpowered to analyze early-onset IS. Therefore, the lack of significant findings could be a consequence of the limited statistical power. Finally, we analyzed soluble *α*-Klotho levels only at single time point—at admission to the hospital with IS signs and symptoms. It would be of interest to measure changes in soluble *α*-Klotho levels in the intervals after IS in a large multiethnic cohort.

## 6. Conclusion

We did not demonstrate the protective role of soluble *α*-Klotho levels with respect to the severity of neurological deficits and long-term outcomes in patients with IS. No neuroprotective effect of soluble *α*-Klotho levels in patients with IS was demonstrated.

## Figures and Tables

**Table 1:** Patient characteristics depending on plasma-soluble *α*-Klotho concentrations.

	<487.7 (*N* = 72)	487.7–813.5 (*N* = 74)	≥813.5 (*N* = 71)
Soluble *α*-Klotho tertiles			
Soluble *α*-Klotho (pg/mL)	336.1 (200.7–420.3)	636.4 (581.2–725.6)	1055.7 (915.5–1271.7)
Age (years)	72 ± 11	71 ± 11	72 ± 11
Sex (men/women)	33/39	35/39	31/40
Previous stroke or transient ischemic attack (*n* (%))	12 (16.7)	16 (21.6)	14 (19.7)
Concomitant diseases			
Hypertension (*n* (%))	63 (87.5)	60 (81.1)	63 (88.7)
Ischemic heart disease (*n* (%))	16 (22.2)	27 (36.5)	20 (28.2)
Atrial fibrillation (*n* (%))	16 (22.2)	23 (31.1)	27 (38.0)
Obesity (*n* (%))	13 (18.1)	9 (12.2)	20 (28.2)^∗^
Diabetes (*n* (%))	27 (37.5)	28 (37.8)	24 (33.8)
Hypercholesterolemia (*n* (%))	35 (48.6)	32 (43.2)	34 (47.9)
Medications before stroke			
Aspirin (*n* (%))	36 (50.7)	36 (48.6)	35 (50.0)
Statin therapy (*n* (%))	32 (44.4)	26 (35.1)	26 (36.6)
Antithrombotic agents (*n* (%))	8 (11.1)	13 (17.6)	6 (8.5)
Addictions			
Active smokers (*n* (%))	17 (23.6)	16 (21.6)	12 (16.9)
Addicted to alcohol (*n* (%))	4 (5.6)	4 (5.4)	2 (2.8)
Type of stroke			
Large vessel occlusion (*n* (%))	41 (56.9)	33 (44.6)	32 (45.1)
Lacunar (*n* (%))	20 (27.8)	20 (27.0)	16 (22.5)
Embolic (*n* (%))	11 (15.3)	21 (28.4)	23 (32.4)
Laboratory findings			
Total cholesterol (mmol/L)	5.19 ± 1.65	5.02 ± 1.31	5.00 ± 1.57
LDL cholesterol (mmol/L)	3.19 ± 1.10	3.26 ± 1.30	3.03 ± 1.17
HDL cholesterol (mmol/L)	1.33 ± 0.36	1.40 ± 0.37	1.44 ± 0.39
Triglycerides (mmol/L)	1.46 (1.12–2.16)	1.34 (0.98–1.82)	1.36 (1.06–1.85)
Creatinine (*μ*mol/L)	80.9 (69.4–92.2)	83.1 (69.7–99.0)	78.5 (70.0–89.3)
Estimated glomerular filtration rate—eGFR (mL/min/1.73 m^2^)	71.0 ± 24.2	68.2 ± 28.4	70.0 ± 21.2
CKD (eGFR < 60 mL/min/1.73 m^2^ or albuminuria) (*n* (%))	26 (36.1)	28 (37.8)	26 (36.6)
C-reactive protein (mg/dL)	5.31 (1.99–13.80)	2.86^∗^ (1.10–8.21)	4.36^∗^ (2.44–8.43)
Calcium (mg/dL)	2.34 (2.22–2.46)	2.29 (2.15–2.46)	2.29 (2.20–2.44)
Phosphorus (mg/dL)	1.09 (0.97–1.26)	1.11 (0.95–1.36)	1.16 (1.01–1.48)
Intact parathyroid hormone (pg/mL)	42.1 (28.8–60.6)	35.9 (22.0–59.6)	41.7 (29.7–56.3)
Intact fibroblast growth factor 23—iFGF23 (pg/mL)	40.3 (16.6–84.3)	50.2 (12.6–83.6)	66.4 (34.0–92.2)
Klotho/iFGF23	6.8 (2.9–21.5)	13.6 (8.3–51.9)	18.8 (10.1–32.7)
Overall survival (OS) and stroke-free survival (SFS)			
12-month OS (*n* (%))	60 (83.3)	55 (74.3)	51 (71.8)
24-month OS (*n* (%))	57 (79.2)	46 (62.2)	47 (66.2)
36-month OS (*n* (%))	45 (62.5)	44 (59.5)	39 (54.9)
12-month SFS (*n* (%))	60 (83.3)	55 (74.3)	51 (71.8)
24-month SFS (*n* (%))	57 (79.2)	45 (60.8)^∗^	46 (64.8)
36-month SFS (*n* (%))	44 (61.1)	43 (58.1)	36 (50.7)

Mean ± standard deviation or median (lower quartile-upper quartile). ^∗^*p* < 0.05.

**Table 2:** Comparison of survivors and patients who died during the period of follow-up.

	Survivors (*N* = 128)	Death (*N* = 89)	*p*
Age (years)	69 ± 10	76 ± 12	<0.001
Sex (men/women)	59/69	40/49	0.87
Previous stroke or transient ischemic attack (*n*/%)	24 (18.7)	18 (20.2)	0.79
Concomitant diseases			
Hypertension (*n* (%))	109 (85.2)	77 (86.5)	0.78
Ischemic heart disease (*n* (%))	31 (24.2)	32 (36.0)	0.06
Atrial fibrillation (*n* (%))	31 (24.2)	35 (39.3)	<0.05
Obesity (*n* (%))	24 (18.8)	18 (20.2)	0.79
Diabetes (*n* (%))	42 (32.8)	37 (41.6)	0.19
Hypercholesterolemia (*n* (%))			
Medications before stroke			
Aspirin (*n* (%))	28 (45.7)	49 (55.7)	0.15
Statin therapy (*n* (%))	47 (36.7)	37 (41.6)	0.47
Antithrombotic agents (*n* (%))	15 (11.7)	12 (13.5)	0.70
Addictions			
Active smokers (*n* (%))	31 (24.2)	14 (15.7)	0.13
Addicted to alcohol (*n* (%))	4 (3.1)	6 (6.7)	0.32
Type of stroke			
Large vessel occlusion (*n* (%))	62 (48.4)	44 (49.4)	<0.05
Lacunar (*n* (%))	41 (32.1)	15 (16.9)	
Embolic (*n* (%))	25 (19.5)	30 (33.7)	
Stroke severity			
NIHSS-I score	7.3 ± 5.2	11.5 ± 7.1	<0.001
Laboratory findings			
Total cholesterol (mmol/L)	5.22 ± 1.62	4.85 ± 1.31	0.07
LDL cholesterol (mmol/L)	3.33 ± 1.33	2.98 ± 1.11	<0.05
HDL cholesterol (mmol/L)	1.38 ± 0.36	1.43 ± 0.51	0.44
Triglycerides (mmol/L)	1.59 (1.15–2.28)	1.23 (0.96–1.56)	<0.001
Creatinine (*μ*mol/L)	78.9 (69.0–89.2)	84.4 (72.9–99.1)	0.08
Estimated glomerular filtration rate—eGFR (mL/min/1.73 m^2^)	72.9 ± 24.4	65.0 ± 24.6	<0.05
CKD (eGFR < 60 mL/min/1.73 m^2^ or albuminuria) (*n* (%))	41 (32.0)	39 (43.8)	0.08
C-reactive protein (mg/dL)	3.82 (1.60–9.21)	4.22 (2.09–9.36)	0.30
Calcium (mg/dL)	2.30 (2.18–2.45)	2.29 (2.20–2.45)	0.69
Phosphorus (mg/dL)	1.13 (0.98–1.38)	1.11 (0.93–1.32)	0.62
Intact parathyroid hormone (pg/mL)	37.3 (22.8–54.2)	42.6 (29.7–65.1)	0.08
Intact fibroblast growth factor 23—iFGF23 (pg/mL)	54.3 (17.6–86.2)	51.5 (25.3–84.9)	0.48
Soluble *α*-Klotho (pg/mL)	627.1 (386.6–870.2)	689.3 (470.8–983.6)	0.18
Klotho/iFGF23	11.5 (6.5–30.5)	14.4 (7.7–26.7)	0.89

Mean ± standard deviation or median (lower quartile-upper quartile).

## Data Availability

The data that support the findings of this study are available from the corresponding author (K.W.) upon reasonable request.
